# Visual Perception, Fine Motor, and Visual-Motor Skills in Very Preterm and Term-Born Children before School Entry–Observational Cohort Study

**DOI:** 10.3390/children7120276

**Published:** 2020-12-05

**Authors:** Anne-Kathrin Dathe, Julia Jaekel, Julia Franzel, Thomas Hoehn, Ursula Felderhoff-Mueser, Britta M. Huening

**Affiliations:** 1 Department of Paediatrics I, Neonatology, Paediatric Intensive Care and Paediatric Neurology, University Hospital Essen, University of Duisburg-Essen, 45122 Essen, North-Rhine Westphalia, Germany; ursula.felderhoff@uk-essen.de (U.F.-M.); britta.huening@uk-essen.de (B.M.H.); 2 Department of Child and Family Studies and Department of Psychology, University of Tennessee, Knoxville, TN 37996, USA; jjaekel@utk.edu; 3 Department of General Paediatrics, Neonatology and Paediatric Cardiology, University Hospital Duesseldorf, 40225 Duesseldorf, North-Rhine Westphalia, Germany; julia.franzel@med.uni-duesseldorf.de (J.F.); thomas.hoehn@uni-duesseldorf.de (T.H.)

**Keywords:** visual perception, fine motor, visual-motor, very preterm, term-born children, preschool age

## Abstract

Very preterm children (<32 weeks gestation at birth; VP) are at risk of developmental difficulties. Specific functional difficulties and delays in visual perception, fine motor, and visual-motor skills have received little research attention, although they are critical for daily life and school readiness. Our aim was to assess these skills in a contemporary cohort of 60 VP and 60 matched term-born children before school entry. We administered the Movement Assessment Battery for Children (M-ABC-2) and the Developmental Test of Visual Perception (DTVP-2). Linear and logistic regressions were run to test group differences in performance and rates of developmental delay in visual perception, fine motor, and visual-motor skills. Very preterm children had lower scores than term-born children in visual perception (*β* = −0.25; *p* = 0.006), fine motor (*β* = −0.44; *p* < 0.001), and visual-motor tasks (*β* = −0.46; *p* < 0.001). The rate of developmental delay (<−1 SD) was higher among VP in visual perception (odds ratio (OR) = 3.4; 95% confidence interval (CI 1.1–10.6)), fine motor (OR = 6.2 (2.4–16.0)), and visual-motor skills (OR = 13.4 (4.1–43.9)) than in term-born controls. VP children are at increased risk for clinically relevant developmental delays in visual perception, fine motor, and visual-motor skills. Following up VP children until preschool age may facilitate early identification and timely intervention.

## 1. Introduction

Survival rates of very preterm infants have increased over the past decades [1,2]. However, while the prevalence of severe disabilities such as cerebral palsy has decreased [3], the high risk of developmental delay in very preterm cohorts has remained unchanged [1,4]. Relatedly, prevalence of severe brain injury after very preterm birth has decreased but diffuse white matter abnormalities have remained stable or increased, causing very similar neurobehavioural consequences [5]. In addition to known difficulties in cognition and executive function in very preterm children [6,7], deficits in motor skills have been documented [8,9,10].

Compared with other areas of developmental difficulties after preterm birth, specific functional impairments in visual-motor skills have received less research attention. However, even subtle developmental delays in fine motor skills and handwriting may negatively affect children’s school performance [11,12] and daily functioning [8]. Studies have reported delays in fine motor skills, visual-motor skills, and visual perception in very preterm children born in the last decades [8,13]. However, these previous studies mainly focused on motor or visual outcomes but did not combine assessments of specific visual perception, fine motor, and visual-motor skills. In general, studies have documented that developmental delays after very preterm birth tend to exacerbate as children grow older [11] and specific demands increase, for example with their entry into formal schooling [14]. Early identification of developmental delay is critical to facilitate timely intervention and support [11,15,16]. In addition to differences between preterm and full-term children, visual perception, fine motor, and visual-motor skills may differ between boys and girls [12,17,18,19] according to the level of parental education [20,21], thus both variables are considered as confounders in the current study.

To assess the extent of delays in visual-motor skills among preterm survivors born in the last decade, we examined very preterm and term-born children at the age of 5 or 6 years, directly before their formal school entry in Germany. We hypothesised that very preterm children would show lower average performance (hypothesis 1) and higher rates of developmental delay (hypothesis 2) in visual perception, fine motor, and visual-motor skills than term-born controls, after controlling for parental education and child sex.

## 2. Materials and Methods

### 2.1. Participants

**Figure 1:**
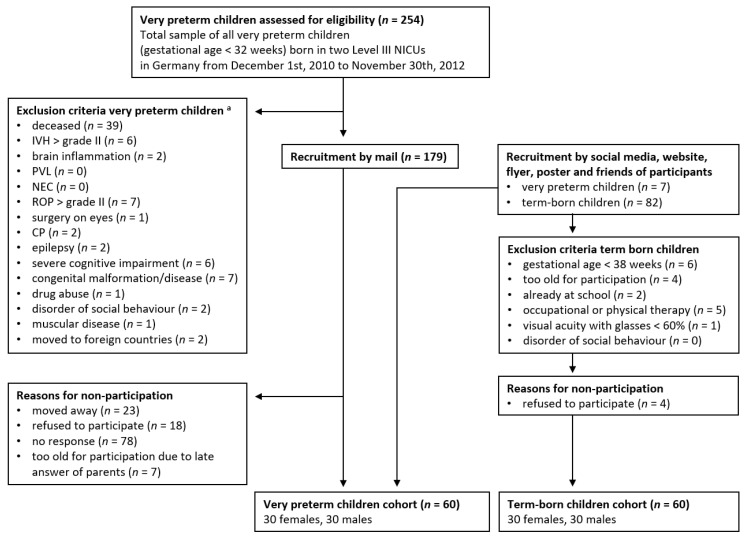
Recruitment strategy and exclusion criteria. Notes. NICU = neonatal intensive care unit, IVH = intraventricular haemorrhage, PVL = periventricular leukomalacia, NEC = necrotising enterocolitis, ROP = retinopathy of prematurity, CP = cerebral palsy, severe cognitive impairment based on diagnoses reported in medical records or defined by intelligence quotient <70, ^a^ participants can fulfill multiple exclusion criteria.

Between June 2017 and August 2018, very preterm (<32 weeks gestation, *n* = 60) and term-born (≥38 weeks gestation, *n* = 60) children’s visual perception, fine motor, and visual-motor skills were assessed in this observational cohort study at mean age 5 years 11 months. Very preterm participants were born between December 2010 and November 2012 in Level III neonatal intensive care units (NICU) in Germany. A summary of the recruitment strategy and exclusion criteria is presented in Figure 1. Children with severe perinatal complications were excluded. A comparison of the very preterm participants’ and non-participants’ perinatal medical records showed that they did not significantly differ with regard to gestational age, birth weight, and child sex (*p* > 0.05). The very preterm and term-born cohorts were stratified by child sex (30 females per cohort). Dropouts and incomplete data of participants did not occur with regard to primary variables.

### 2.2. Standard Protocol Approvals and Patient Consents

The study was carried out in accordance with the Helsinki Declaration and approved by the local ethical committees in Essen (16-7265-BO) and Duesseldorf (2017074357). Parents of all participants gave written informed consent and children gave verbal assent for participation and publication.

### 2.3. Clinical Characteristics

Perinatal clinical characteristics were collected from infant medical records. Gestational age (weeks, range: very preterm = 23 + 6 – 31 + 6, term comparisons = 38 + 0 – 42 + 0), birth weight and sex were assessed at birth. Intraventricular haemorrhage (IVH) was determined via ultrasound examination and classified into grade I, II, III or parenchymal haemorrhagic infarction [22]. Bronchopulmonary dysplasia (BPD) was defined by the need for supplemental oxygen at 36 weeks postmenstrual age. BPD was classified into mild (respiratory support without supplemental oxygen), moderate (with <30% O_2_) or severe (≥30% O_2_ and/or positive pressure) [23]. Persistent ductus arteriosus (PDA) was identified by echocardiography [24]. Sepsis was defined as a systematic inflammatory response syndrome in the presence of suspected or proven infection or as a result of them [25]. The diagnosis of retinopathy of prematurity (ROP) was based on international ROP classification [26].

### 2.4. Parental Education

The highest educational qualification held by either parent was obtained via questionnaire and coded according to the International Standard Classification of Education (ISCED) into low (level 0–2), middle (level 3–4), and high (level 5–8) [27]. The category “low” was not present, resulting in a binary-coded variable parental education (medium vs. high).

### 2.5. Outcome Measures

Visual-motor skills were assessed with the German version of the Developmental Test of Visual Perception 2 (DTVP-2) [28,29], encompassing four motor-enhanced subtasks: Eye-Hand Coordination (drawing straight lines); Copying (drawing different geometrical shapes); Spatial Relation (connecting different points by drawing straight lines according to a template); and Visual–Motor Speed (assigning two different symbols to a set of geometrical shapes within one minute). Visual perception, in particular Position in Space (distinguishing the spatial orientation of different shapes), was also evaluated using the DTVP-2. The DTVP-2 has good reliability (Cronbach *α* = 0.78–0.88 for subscales) and high validity (correlations with the Beery–Buktenica Developmental Test of Visual-Motor Integration (VMI) and the Test of Visual Perceptual Skills – Revised (TVPS-R) subscale scores >0.70) [28,30,31]. Fine motor skills were measured using the subtask Manual Dexterity of the Movement Assessment Battery for Children - second edition, German version (M-ABC-2) [32,33]. The subtask contains threading beads, dropping coins into a slot, and guiding a pen within lines. The M-ABC-2 is one of the most popular motor assessments [34,35], with acceptable reported validity and reliability [32].

For both assessments, raw scores of each subtest were transformed into age-adjusted standard scores with a mean (SD) of 10 (3), higher scores indicating better performance. Standard scores lower than 1 SD of the normative mean (i.e., <7) are an indication of a developmental delay and were binary-coded accordingly [28,32].

### 2.6. Statistical Analysis

Analyses were performed using SPSS 23 (IBM Corp, New York, NY, USA). Linear regressions were run to evaluate group differences in visual perception, fine motor, and visual-motor skills (step 1). Child sex and parental education were entered as control variables in step 2. Analyses were repeated using logistic regressions to determine group differences with regard to risk of developmental delay. We used Bonferroni correction to avoid inflation of type I error for multiple testing by adjusting the alpha level to *p* = 0.025.

Sample size estimation was performed a priori (before starting data collection), using the Optimal Design software. Based on an expected effect size of *d* = 0.62 [9] for the difference in the continuously distributed primary outcome fine motor skills between very preterm and term children, an alpha level of *p* = 0.025, and an estimated 5% of explained variance by the control variables child sex and parental education, the required total sample size was *N* = 98 children (*n* = 49 per group) to achieve 80% power for multiple linear regression. Subgroup analyses are not necessary with regard to the hypotheses.

## 3. Results

**Figure 2:**
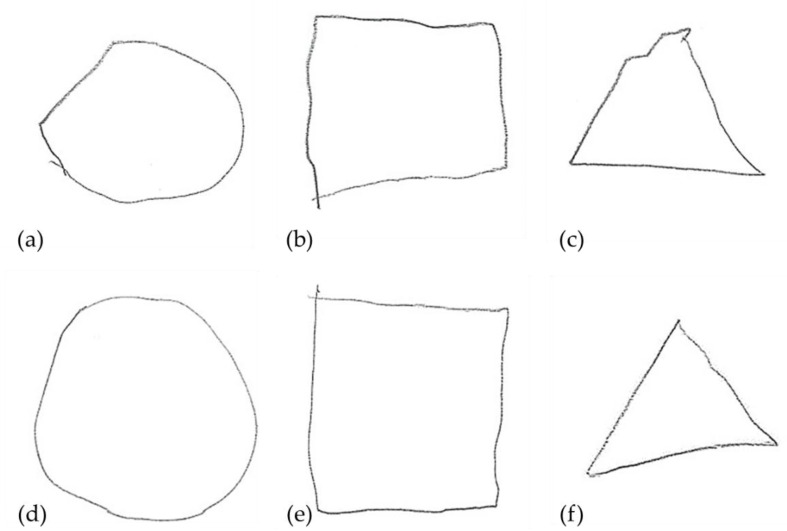
Example comparison of geometrical shapes drawn by a very preterm (**a**–**c**) and term-born child (**d**–**f**).

Descriptive findings in Table 1 show that very preterm children were born at lower gestational ages and birth weights than the term-born controls. No group differences existed with regard to child sex, age at assessment, and parental education. Very preterm infants (*n* = 60) suffered from more medical complications (*n* = 4 IVH grade I/II, *n* = 13 ROP grade I/II, *n* = 1 mild BPD, *n* = 27 PDA, *n* = 12 sepsis) than term-born controls (*n* = 1 sepsis). On average, very preterm children showed weaker performance in visual perception, fine motor, and visual-motor skills than term-born controls with moderate-to-high effect sizes (*d* = 0.53, *p* = 0.004; *d* = 0.98, *p* < 0.001; *d* = 1.1, *p* < 0.001). Example differences in drawing geometrical shapes (i.e., visual-motor skills) are shown in Figure 2. The first set of drawings of a very preterm child (a–c) shows several askew and broken lines; in contrast, the second set of drawings from an age- and sex-matched term-born child (d–f) shows relatively even and solid lines. These differences are reflected in the coded standard scores of the subtask copying. 

**Figure 3:**
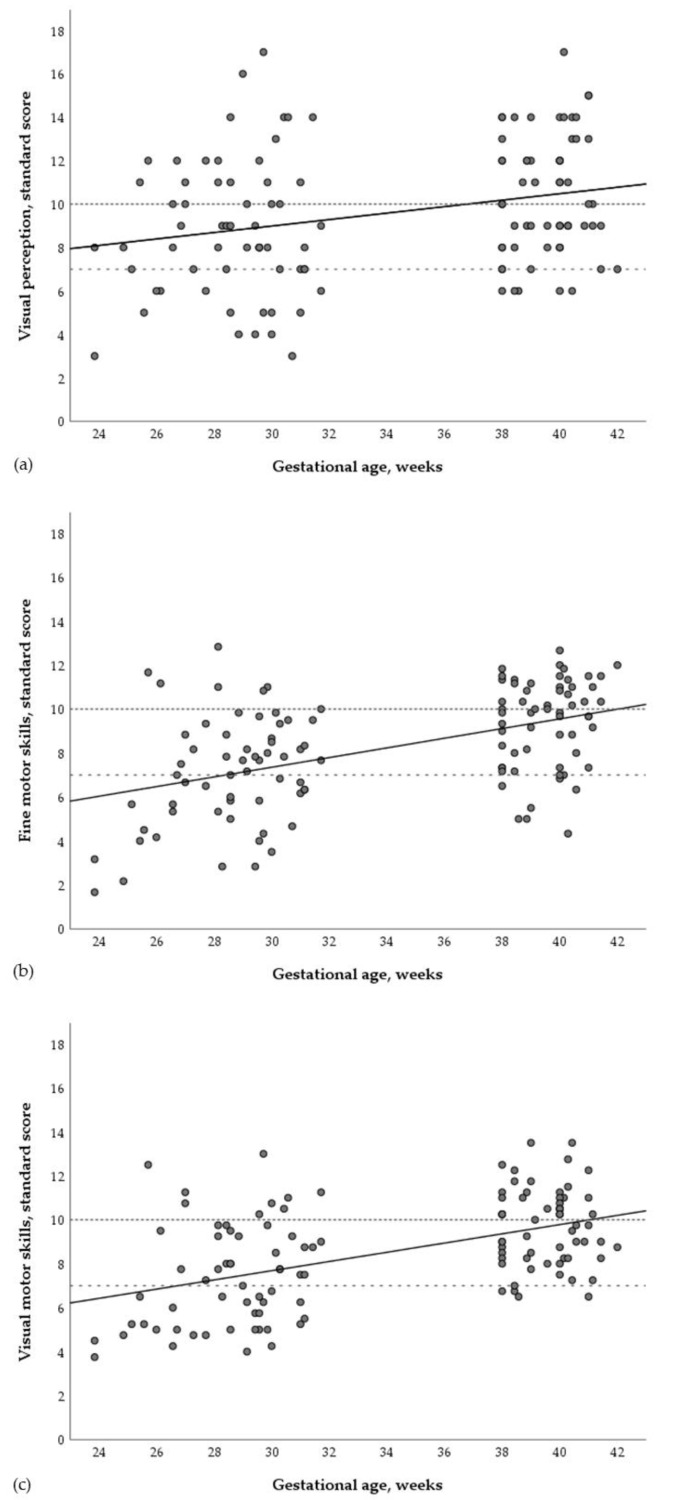
Distributions (scatter plot) and simple linear regression lines showing associations of (**a**) visual perception, (**b**) fine motor, and (**c**) visual-motor skills with gestational age at birth. Notes. Dotted horizontal lines show each tests’ normed standard scores of 10 (i.e., the baseline mean) and the normed cutoff scores of 7 (i.e., −1 standard deviation below the baseline), indicating clinical developmental delay.

The distribution of individual data points with regard to the association of visual perception, fine motor, and visual-motor skills with gestational age at birth is shown in Figure 3. Linear regressions confirmed hypothesis 1, i.e., very preterm children were less skilled in visual perception (*β* = −0.25; *p* = 0.006), fine motor (*β* = −0.44; *p* < 0.001), and visual-motor tasks (*β* = −0.46; *p* < 0.001) than term-born controls (Table 2), even after controlling for confounders.

Hypothesis 2 was also confirmed: The risk of clinically significant developmental delay (<−1 SD) was higher among very preterm children in visual perception (odds ratio (OR) = 3.4; 95% confidence interval (CI) (1.1–10.6)), fine motor (OR = 6.2 (2.4–16.0)), and visual-motor skills (OR = 13.4; (4.1–43.9)) than in term-born controls (Table 3; also see Figure 3 for details about individual participants scoring below the clinical cutoff score of 7 in each group). All effects persisted after adjusting for parental education and child sex.

## 4. Discussion

This study represents the first combined investigation of group differences between very preterm-born children and matched term-born controls in visual perception, fine motor, and visual-motor skills at the age of 5 and 6 years in a recent cohort. Both hypotheses were confirmed: very preterm children showed statistically significant lower performance (hypothesis 1) and a higher risk of developmental delays (hypothesis 2) in visual perception, fine motor, and visual-motor skills than their term-born peers. Group differences remained stable even when controlled for child sex and parental education.

These findings are in line with existing meta-analyses, where very preterm children showed lower performance. However, in the current study of infants born in the last decade, effect sizes were higher in visual perception (*d* = 0.53 vs. *d* = 0.42), fine motor (*d* = 0.98 vs. *d* = 0.62), and visual-motor skills (*d* = 1.1 vs. *d* = 0.69) than previously reported [9,13,36]. These findings are particularly remarkable in light of the homogeneous and low-risk characteristics of this cohort of very preterm children. Accordingly, very preterm birth itself may be regarded as a risk factor for visual perception, fine motor, and visual-motor skill development. The significant developmental differences found here may be explained by a narrower age range, homogeneous neurological risk profile, and the inclusion of a control group compared to previously published pooled meta-analytical data. Furthermore, there are various methods of assessment for visual-motor skills. The VMI, for instance, reported in the meta-analysis by Geldof and colleagues [13], includes only the task Copying, whereas the DTVP-2 has additional subtests. Interestingly, very preterm children showed lower performance in Copying as measured by VMI, as well as in the subtest Spatial Relationship (by connecting points according to a template) and in Eye–Hand Coordination (exact guidance of a pen within lines).

In standardised fine motor tests, the duration and number of errors for specific tasks are recorded. In the current study, very preterm children required on average more time and more trials to complete a task. Marlow and colleagues supplemented these findings by reporting more associated movements and dysfunction in posture in very preterm infants compared to controls [10]. It can only be assumed what consequences these qualitative observations mean for daily life and school situations. Ecologically valid assessments, including environmental factors of everyday activities, are required to reflect the relevance of difficulties or developmental delays. 

In the present study, the potential confounders child sex and parental education did not have significant effects on the assessed skills. The impact of sex on the development of special abilities such as fine motor skills is discussed. In peg board and threading tasks, girls are described as being more skillful and faster than boys [37], whereas almost no sex differences are known in standardised tests such as the M-ABC-2 [17,32]. Studies report that lower visual-motor skills are more often found in boys than in girls [13,37,38]. This may be related to the use of the VMI test, because gender differences were not taken into account in the test standardisation. In contrast, the German version of the DTVP-2 has gender-specific normative data with lower requirements for boys, which were applied here and might explain the nonsignificant effects of gender in the present study. The impact of parental education on visual perception, fine motor and visual-motor skills, however, has previously not been investigated and replication of our nonsignificant findings is warranted.

Although there is vast evidence of functional neural aberrations after very preterm birth, the specific pathogenesis of developmental delay in visual perception, fine motor, and visual-motor skills is not yet fully understood. Altered brain structures and networks are discussed since MRI imaging, apart from severe focal lesions, has shown volume reduction of the white and grey matter [39] and reduced microstructural integrity of the white structure [40,41]. In an explorative study, Bolk and colleagues described a positive correlation of the precentral gyrus volume at term equivalent age with fine motor and visual-motor skills in infants born extremely preterm at the age of six years [15]. Furthermore, fine motor skills were positively correlated with cerebellar and brainstem volume [15], suggesting that these regions likely play a role in the development of the developmental dimensions investigated here. In an innovative MRI examination, white matter microstructure was examined by Young et al. using Diffusion Tensor Imaging (DTI). They reported higher directed diffusion (fractional anisotropy) of water molecules parallel to white matter tracts among term-born compared to very preterm children at the age of six years [40]. Furthermore, a correlation between low fractional anisotropy or low Neurite Density Index (NDI) and poorer results in intelligence and visual-motor tests in very preterm infants was reported. The authors described the reduced NDI as a measure of changes in axonal structure as well as in myelin [40]. This study provides first indications of microstructural alterations in underlying neuronal networks involved in visual-motor function. Due to resource constraints, there was no neuroimaging at the time of testing in the present study. However, very preterm children with severe lesions on neonatal ultrasound scans were excluded.

Very preterm children are at increased risk for various disorders and lower academic achievement compared to their healthy term-born peers [42,43]. Disorders of visual perception, fine motor, and visual-motor skills usually become evident in middle childhood, in a formal school context, when specific demands arise and differences between classmates become apparent. In particular, the relevance of visual-motor skills increases rapidly in the first year of school due to the critical acquisition of handwriting skills [12,44]. Furthermore, fine motor and visual-motor tasks play a major role in school and take up 31% to 60% of everyday school life [45]. For the recognition and reading of letters, visual perception plays an essential role. Studies of very preterm children at school have shown that low visual perception performance correlates with poor reading skills [46,47]. Therefore, early identification of children at risk for delays may open a time window for intervention before school entry. However, there is currently no specific intervention with proven efficacy due to the lack of valid, randomised controlled studies in preschool populations [16]. Future research should not only investigate associations between visual perception, fine motor, and visual-motor skills, but also focus on the evaluation of specific interventions and their potential long-term effects.

Strengths of the current study are a homogeneous cohort of low-risk very preterm children born post-2010, the combined investigation of visual perception, fine motor, and visual-motor skills and controlling of results for the confounders parental education and child sex. Previous studies did not investigate all three skills simultaneously, thus lacking documentation of a complete developmental profile, or they did not include a control group [8,9,13,15]. However, there are also limitations. The current results may be affected by a high rate of families not responding to the study invitation, maybe due to frequent moves in an urban area, and the semi-structured recruitment of term-born children. The families who agreed to participate may thus be of higher socioeconomic status than representative for the study catchment area, also indicated by the absence of parents with low educational levels. Nevertheless, parental education did not differ between groups; therefore, only a small bias is assumed for the size of this effect, possibly an underestimation due to limited variance. In addition to differences in parental education, children’s daily screen times and use of smartphones or tablets might have an effect on fine motor and visual-motor skills in typically developing children [48] and may be addressed in future studies. Due to time and cost constraints, the protocol did not include neuroimaging; therefore, the current study cannot expand the knowledge about association between developmental delay and cerebral changes. It seems reasonable to investigate various brain network structures, e.g., in functional MRI studies, in order to identify protective and disruptive factors. Finally, we were not able to measure parental support in drawing, manual dexterity and visual perception. 

In conclusion, very preterm children had lower skills and higher rates of developmental delay in visual perception, fine motor, and visual-motor skills than term-born controls. Children with such difficulties may show tense finger postures, require more time to complete daily fine motor activities, and have to focus on motor performance at the expense of cognitive function. Future research should focus on the underlying mechanisms of visual perception, fine motor, and visual-motor skills, their potential precursors, and the particular importance of handwriting skills. Timely evidence-based interventions need to be developed and evaluated for effectiveness.

## Figures and Tables

**Table 1:** Descriptive characteristics of the very preterm and term participant groups.

	Very Preterm (*n* = 60)	Term (*n* = 60)	*p* ^a^
**Clinical characteristics**			
Gestational age, weeks (range)	28.7 (23.9–31.7)	39.5 (38.0–42.0)	<0.001
Birth weight, gram (range)	1126.0 (430–1860)	3414.3 (2380–4895)	<0.001
Female, *n*	30	30	1.0
**Follow-up characteristics**			
Age at assessment, years	5.9 (0.3)	5.9 (0.3)	0.681
Parental education (high, *n* (%))	36 (60)	41 (68)	0.341
Visual perception, standard score	8.8 (3.2)	10.4 (2.8)	0.004
Fine motor skills, standard score	7.1 (2.5)	9.4 (2.0)	<0.001
Visual-motor skills, standard score	7.4 (2.3)	9.6 (1.8)	<0.001

Notes. Data are presented as mean (standard deviation) if not indicated otherwise. ^a^ *T*-test and Chi-square results for continuous and non-continuous data, respectively.

**Table 2:** Regression analysis investigating effects of very preterm birth, child sex, and parental education on visual perception, fine motor, and visual-motor skills (*n* = 120).

	Visual Perception				Fine Motor Skills				Visual-Motor Skills			
	*b*	95% CI	*β*	*p*	*b*	95% CI	*β*	*p*	*b*	95% CI	*β*	*p*
**Step 1**												
Very preterm	−1.60	−2.68 to −0.52	−0.26	0.004	−2.23	−3.06 to −1.40	−0.44	<0.001	−2.24	−2.99 to −1.49	−0.48	<0.001
Total R^2^		0.07				0.20				0.23		
**Step 2**												
Very preterm	−1.53	−2.61 to −0.45	−0.25	0.006	−2.19	−3.02 to −1.37	−0.44	<0.001	−2.17	−2.91 to −1.43	−0.46	<0.001
Female	−0.11	−1.21 to 0.99	−0.02	0.841	0.59	−0.26 to 1.42	0.12	0.170	−0.10	−0.86 to 0.66	−0.02	0.793
Education high	0.85	−0.30 to 2.00	0.13	0.147	0.45	−0.43 to 1.33	0.09	0.316	0.91	0.12 to 1.70	0.19	0.025
Total R^2^ (∆ R^2^) ^a^		0.09 * (0.02)				0.21 *** (0.02)				0.27 *** (0.04)		

Notes. CI = confidence interval; Education = parental education. ^a^ Differences between Total R^2^ and the sums of ∆ R^2^ are due to rounding; negative regression-coefficients indicate lower skills, statistical significance of explained variance R^2^ based on F test. * *p* < 0.05. *** *p* < 0.001.

**Table 3:** Developmental delay (<−1 SD, %, OR) in visual perception, fine motor, and visual-motor skills in very preterm (*n* = 60) and term children (*n* = 60).

	Very Preterm	Term	Unadjusted OR (95% CI)	Adjusted OR ^a^ (95% CI)
Visual perception	23.3	8.3	3.35 (1.12–9.99)	3.45 (1.12–10.64)
Fine motor skills	45.0	11.7	6.20 (2.43–15.83)	6.24 (2.42–16.04)
Visual-motor skills	46.7	6.7	12.25 (3.94–38.08)	13.48 (4.13–44.00)

Notes. SD = standard deviation; OR = odds ratio; CI = confidence interval. ^a^ Adjusted for parental education and child sex.
